# Tissue Reaction and Biocompatibility of Implanted Mineral Trioxide Aggregate with Silver Nanoparticles in a Rat Model

**DOI:** 10.7508/iej.2016.01.003

**Published:** 2015-12-24

**Authors:** Vahid Zand, Mehrdad Lotfi, Amirala Aghbali, Mehran Mesgariabbasi, Maryam Janani, Hadi Mokhtari, Pardis Tehranchi, Seyyed Mahdi Vahid Pakdel

**Affiliations:** a * Dental and Periodontal Research Center, Department of Endodontics, Dental School, Tabriz University of Medical Sciences, Tabriz, Iran;*; b * Research Center for Pharmaceutical Nano-Technology, Department of Endodontics, Dental School, Tabriz University of Medical Sciences, Tabriz, Iran;*; c * Department of Oral and Maxillofacial Pathology, Dental School, Tabriz University of Medical Sciences, Tabriz, Iran;*; d * Researcher, Drug Applied Research Center, Tabriz University of Medical Sciences, Tabriz, Iran;*; e * Department of Endodontics, Dental School, Tabriz University of Medical Sciences, Tabriz, Iran; *; f * Department of Restorative Dentistry, Dental School, Tabriz University of Medical Sciences, Tabriz, Iran; *; g * Department of Prosthodontics, Faculty of Dentistry, Tabriz University of Medical Sciences, Tabriz, Iran*

**Keywords:** Biocompatibility, Mineral Trioxide Aggregate, Nanosilver, Silver Nanoparticle

## Abstract

**Introduction:**

Biocompatibility and antimicrobial activity of endodontic materials are of utmost importance. Considering the extensive applications of mineral trioxide aggregate (MTA) in dentistry and antimicrobial properties of silver nanoparticles, this study aimed to evaluate the subcutaneous inflammatory reaction of rat connective tissues to white MTA with and without nanosilver (NS) particles.

**Methods and Materials:**

Polyethylene tubes (1.1×8 mm) containing experimental materials (MTA and MTA+NS and empty control tubes) were implanted in subcutaneous tissues of seventy-five male rats. Animals were divided into five groups (*n*=15) according to the time of evaluation: group 1; after 7 days, group 2; after 15 days, group 3; after 30 days, group 4; after 60 days and group 5; after 90 days. The inflammatory reaction was graded and data was analyzed using the Kruskal-Wallis and Mann-Whitney U tests. Statistical significance was defined at 0.05.

**Results:**

Comparison of cumulative inflammatory reaction at all intervals revealed that the mean grade of inflammatory reaction to MTA, MTA+NS and control samples were 3, 2 and 2, respectively. According to the Mann-Whitney analysis there were no significant differences between MTA+NS and MTA (*P*=0.42).

**Conclusion:**

Incorporation of 1% nanosilver to MTA does not affect the inflammatory reaction of subcutaneous tissue in rat models.

## Introduction

Mineral trioxide aggregate (MTA) is a mixture of dicalcium silicate, tricalcium silicate, tricalcium aluminate, tetracalcium alumino ferrite and bismuth oxide [1-3]. It also contains other elements such as SiO_2_, CaO, MgO, Al_2_O_3_, K_2_SO_4_, FeO and Na_2_SO_4_ [4]. MTA is used in vital pulp therapy (direct pulp capping, partial pulpotomy and pulpotomy), repair of furcation and lateral canal perforations, as an apical plug in root canal treatment of open apex non-vital teeth and as a root-end filling material during apical surgery [5, 6]. 

Some studies have reported limited antimicrobial effect of MTA against some microorganisms [7-10]. However, it has antibacterial effects against some facultative bacteria and does not affect strict anaerobes.

Silver salts and their derivatives are commercially employed as antimicrobial agents [11]. Silver nanoparticles (nanosilver-NS) are amongst the most widely used nanoparticles for medical applications such as preventing bacterial colonization on catheters, prostheses and clothing [12]. 

However, due to its concentration-dependent toxicity, silver should be used with caution. The small particle size, high surface area per unit mass, chemical composition and surface characteristics might be important in this toxicity mechanism [13]. The bacterial toxicity of NS might be explained by their interaction with bacterium and particle cellular internalization [14, 15]. Size-dependent toxicity of NS can be another mechanism of action [16, 17]. Small-sized particles of NS can inhibit nitrifying bacterial growth which is much more than silver ions at similar concentration [18]. 

**Figure 1:**
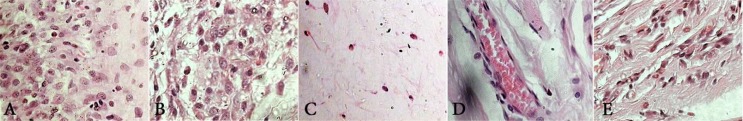
Histologic images of inflammatory cell infiltration (lymphocytes, plasma cells, polymorphonuclear leukocytes and macrophages) at the end of implanted MTA+NS tubes: *A and B)* Grade 3 inflammation was observed at 7- and 15-day intervals; *C)* Grade 2 inflammation was observed at 30-day samples; *D and E)* Grade 1 inflammation was observed at 60- and 90-day intervals (Hematoxylin-Eosin staining) (Original magnification 400×)

Despite the widespread use of NS products, few studies have determined the biological effects of NS on mammalian cells. Most studies have reported the mild tissue reaction to sliver particles, especially in low concentrations [19]. Gomes-Filho *et al.* [20] concluded that NS was biocompatible, especially in low concentrations [20]. 

It seems that increasing antibacterial properties of MTA, especially against *E. faecalis *could be beneficial. *Samiei* et al. *[*21*]* *showed that* adding NS to MTA improved its antimicrobial efficacy against E. faecalis, C. albicans and P. aeruginosa. 

The present animal implantation study aimed to evaluate the subcutaneous inflammatory reaction of rat connective tissues to tooth-colored MTA without and with NS (MTA+NS).

## Materials and Methods

For this study thirty five 2-3 month male Wistar albino rats weighting 250±30 g were randomly selected. All ethical criteria contained in Declaration of Helsinki and the considerations recommended by Institutional Animal Care and Use Committee (IACUC) on the care and use of laboratory animals, were observed in different stages of the project. 

The animals were anesthetized with 5 mg/kg of 2% Xylazine HCL (Alfasan, Woerden, Holland) and then IM injection of 50 mg/kg Ketemine HCL (Rotexmedica, Trittau, Germany). After shaving, three separate 2-cm incisions were prepared on the back of each animal with at least 2 cm distances from each other. Tooth-colored MTA (Dentsply, Tulsa Dental, OK, US) was mixed with distilled water according to the manufacturer’s instructions. Also MTA was mixed with 1% wt of NS (NanoSilver Solution, Lotus Nanochemistry, Pasargad, Tehran, Iran) and then mixed with distilled water at a liquid/powder ratio of 0.3 mL/g. Freshly mixed materials were immediately placed in sterile polyethylene tubes with a 1.1 mm inner diameter and 8 mm length and were subcutaneously implanted in 2 separate incisions. An empty polyethylene tube was implanted in the third incision in each animal as control [22].

The rats were sacrificed at 7-, 15-, 30-, 60- and 90-day intervals by administration of high-dose diethyl-ether in an induction chamber. Then the tubes and surrounding tissues were removed in blocks and fixed in 10% buffered formalin solution for 10 days. The specimens were then processed for routine paraffin embedding. Subsequently, 5 mm-thick tissue sections were prepared longitudinally through the midline of the tubes and stained with Hematoxylin-Eosin. Evaluation of inflammatory cells (lymphocytes, plasmocytes, polymorphonuclear leukocytes, macrophages and giant cells) was done in microscopic fields adjacent to test materials at the end of the tubes under a light microscope (Carl Zeiss, Oberkachen, Germany) under 400× magnification. The average value of inflammatory cells for each specimen was obtained from the sum of cells counted in 4 separate areas [22]. The observer was blinded to the materials used in the specimens. The overall mean value for each material was determined in subjects at each interval. The inflammatory reactions were categorized as *grade 0, *without inflammatory cells; *grade 1*, with mild inflammation (cells <25); *grade 2*, with moderate inflammation (cells=25-125) and *grade 3*, with severe inflammation (>125 cells). The Kruskal-Wallis and Mann-Whitney tests were used for statistical analysis. The level of significance was set at 0.05.

## Results

At 7-day interval, the mean of inflammation grades were 3, 3 and 2 for MTA and MTA+NS and control samples, respectively. The inflammation was consisted of severe infiltration of inflammatory cells (lymphocytes, plasmocytes, polymorphonuclear leukocytes and macrophages) (Figure 1A and 1B) with no significant differences between either test materials (*P*=0.48). 

At 15-day interval, the mean value of inflammation grades were 3 for MTA, MTA+NS and the control groups, respectively, which also consisted of severe infiltration of inflammatory cells (Figure 1A and 1B). There were no significant differences between MTA and MTA+NS (*P*=1).

At 30-day interval, the mean of inflammation grades were 3, 2 and 2 for MTA+NS, MTA and control groups, in order of appearance (Figure 1C). There were no significant differences between MTA and MTA+NS (*P*=0.05). 

**Figure 2:**
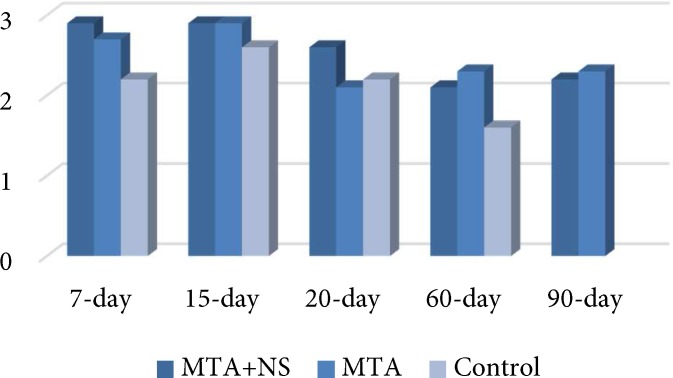
The mean value of inflammation grades in different time intervals

At 60- and 90-day intervals, the mean of inflammation grades were 2 for all groups, which consisted of moderate infiltration of inflammatory cells (Figure 1D and 1E). There were no significant differences between MTA and MTA+NS and between the two experimental groups and the control group (*P*=0.36 and *P*=0.73, respectively) (Figure 2).

## Discussion

In this study the biocompatibility of MTA and MTA+NS was evaluated by subcutaneous implantation of set test materials. The tissue reaction was not significantly different in different groups. This procedure was introduced by Torneck [23] and was approved by Olsson *et al. *[24] who evaluated the tissue reaction after 7-, 15-, 30-, 60- and 90-day intervals. The general consensus is that both short- and long-term inflammatory reactions can be assessed in a single study [25-28]. Indeed, placement of the experimental materials in tubes simulates the clinical conditions [29]. 

At 7- and 15-day intervals, MTA provoked a severe inflammatory reaction in comparison with the control group, which is consistent with the findings of many other studies [22, 25, 26, 30, 31]. Another animal study reported the similarity of connective tissue response after subcutaneous implantation of MTA and calcium hydroxide. Similar to calcium hydroxide implants, areas of coagulation necrosis and dystrophic calcification were observed with MTA implants [31]. The major ingredients of MTA match the main ingredients of Portland cement [4, 32]. Similar to Portland cement, MTA would generate calcium hydroxide when mixed with water which would explain the high pH of the set material and inflammatory reactions subsequent to its subcutaneous implantation [33, 34]. 

Yaltrik *et al.* [22], demonstrated that connective tissue reaction to amalgam and ProRoot MTA was similar at 7-, 15-, 30- and 60-day intervals. In the present study, connective tissue reaction to all the experimental materials was more than that of the control group at all time-intervals. Also the empty control tubes groups provoked less inflammatory reactions, which is similar to the results of previous studies [23, 25, 26, 35, 36].

In the present study, inflammatory reaction in the MTA+NS group at 15-day interval reached a maximum level and followed a decreasing pattern afterwards. This trend indicates the undesirable short-term and desirable long-term reactions that has also been reported by Lotfi *et al*. [26], Vosoughhoseini *et al. *[25] and Holland *et al. *[30]. In addition, the inflammatory reaction induced by MTA+NS was the same as MTA at initial and 60-day and 90-day intervals. 

Regarding cumulative inflammatory reaction, the tissue reactions to MTA and MTA+NS showed no differences. In an *in vitro* study the antimicrobial efficacy of MTA with NS particles (1% weight) was compared to MTA; adding NS to MTA improved its antimicrobial efficacy against *E. faecalis*, *C. albicans* and *P. aeruginosa *[21].

However, silver should be used with caution because of its toxicity which is concentration-dependent. Silver nanoparticles have antibacterial effects as well [19]. Size-dependent toxicity is the main mechanism of action of NS on bacteria [16, 17]. Gomes-Filho *et al.* [20] evaluated the tissue response to implanted polyethylene tubes filled with fibrin sponge embedded with silver nanoparticle dispersion: low concentration of NS (23 ppm) induced a mild reaction, and silver nanoparticle dispersion was generally biocompatible. The same result was obtained in the present study and regarding cumulative inflammatory reaction, the tissue reaction to MTA and MTA+NS in lower concentration (1% wt.) showed no difference, demonstrating the biocompatibility of this combination. On the other hand adding NS to MTA may cause some physical and chemical alterations in this material that need to be assessed.

## Conclusion

The inflammatory reaction induced by MTA with and without silver nanoparticles was similar in the *in vivo* setting. Therefore, conducting further research to assess other physical and chemical alterations in this mixture, is suggested.
